# Anxiety and its effect on foreign medical students

**DOI:** 10.1192/j.eurpsy.2025.1012

**Published:** 2025-08-26

**Authors:** N. Bouattour, F. Guermazi, R. Jbir, D. Mnif, M. Lalaoui Rhali, I. Baâti, J. Masmoudi

**Affiliations:** 1 Psychiatry A, Hedi Chaker University Hospital, Sfax, Tunisia

## Abstract

**Introduction:**

Nowadays thousands of students choose to get into medical universities in foreign countries for a plethora of reasons. Tunisia represents one of these destinations, thanks to its geographic localization, weather, biological diversity as well as its ethnical and cultural background.

**Objectives:**

To determine the effect of anxiety and psychological distress on the motivation of studies in foreign students in Tunisia.

**Methods:**

We conducted a cross-sectional, descriptive and analytical study, carried out between July and September 2023 among foreign students at the Sfax Faculty of Medicine. Data collection was carried out using an anonymous self-questionnaire via “Google Forms shared via social media. Psychological distress was assessed using the “DASS-21” Depression, Anxiety and Stress Scale. Motivation was assessed using the French version of the “SMMS-R-FR” Study Motivational Strength Questionnaire.

**Results:**

Seventy-two foreign medical students completed the survey. The average age was 25 ± 3.45 years. The majority of students were male (57%). Coffee and tobacco were the most consumed substance by the students (88.9%, and 47.2% respectively). The mean score for score anxiety score was 6.59, 7.2 for depression and 7.83 for stress. The mean score for the strength of motivation was 42.4. We found a negative statistical association between the anxiety dimension and the strength of motivation (p=0.011, r= -0.29). We found no statistical association with the depressive symptoms dimension nor with the stress dimension.Smoking was correlated with anxiety and depression in our foreign students (p=0.02 and p=0.027 respectively).

**Conclusions:**

Identifying psychological distress and screening for them can help prevent the deterioration of mental health of foreign students and therefore the deterioration their academic performance.

**Disclosure of Interest:**

None Declared

